# Selection and evaluation of citrus phloem specific promoters

**DOI:** 10.1186/1753-6561-8-S4-P99

**Published:** 2014-10-01

**Authors:** Laura Melissa Gómez-Krapp, Polyana Kelly Martins, Francisco de Assis Alves Mourão Filho, Liliane Cristina Liborio Stipp, Lísia Borges Attílio, Rodrigo Guarino Cassarotti, Marcos Antônio Machado, Juliana Freitas-Astúa

**Affiliations:** 1 Centro de Citricultura Sylvio Moreira, Instituto Agronômico de Campinas, Cordeirópolis, SP, Brazil; 2 EMBRAPA Agroenergy - Estação Parque Biológico, Brasilia, DF, Brazil; 3 Escola Superior de Agricultura "Luiz de Queiroz", Universidade de São Paulo, Piracicaba, SP, Brazil; 4 Escola Superior de Agricultura "Luiz de Queiroz", Universidade de São Paulo, Piracicaba, SP, Brazil Centro de Citricultura Sylvio Moreira, Instituto Agronômico de Campinas, Cordeirópolis, SP, Brazil; 5 EMBRAPA Cassava & Fruits, Cruz das Almas, Bahia, Brazil Centro de Citricultura Sylvio Moreira, Instituto Agronômico de Campinas, Cordeirópolis, SP, Brazil

## Background

The citrus industry worldwide has faced major economic losses due to the occurrence of *Huanglongbing *(HLB), a disease caused by *Candidatus *Liberibacter spp., Gram-negative bacteria restricted to the phloem of their hosts. Due to the lack of commercial varieties that could resist to these bacteria, genetic transformation comes as an important alternative for improving these varieties and managing the disease. Most of the work with citrus genetic transformation uses constitutive promoters, but recently studies have intensified the search for tissue-specific promoters, making possible to direct the transgene expression to the phloem and reduce it in other undesired parts of the plant, such as the fruits. It is possible that tissue-specific promoter sequences obtained from the same plant species have better acceptance by the customers and this was the objective of our work: prospect phloem-specific promoters from the citrus genome that can be used for citrus transformation with the objective to deliver transgene expression directly to the tissue of *Ca*. Liberibacter spp. accumulation.

## Methods

Promoters prospection was made from available citrus EST databases (CitEST/HarvEST, available locally), and from the complete genome sequences of *Citrus sinensis *and *C. clementina *(available at http://www.phytozome.net). Five selected sequences, identified with the program PLACE (http://www.dna.affrc.org.jp/place/), were cloned into pCambia 2301 containing the *uid*A gene and inserted into *Agrobacterium tumefaciens *EHA 105. The genetic transformation was performed according to Miyata *et al*. [1].

## Results and conclusions

Epicotyl segments obtained from Carrizo citrange and Hamlin sweet orange seedlings were used as source of explants for genetic transformation. From each promoter, three independent experiments were made. In each experiment an average of 300 explants were introduced and approximately 200 buds regenerated. The GUS histochemical test was performed and confirmed the transformation of 2 to 24% positive plants, depending on the construction. The preliminary results exhibit preferential expression in phloem cells. The transgene presence is being confirmed by PCR. A comparison among the GUS expression induced by the promoters will be conducted in order to determine which of them is more efficient in driving the transgene expression to the phloem cells. Such promoter will be further used in expression cassettes constructed with candidate genes for the control of HLB.
